# Clinical Evaluation of Effects of Chronic Resveratrol Supplementation on Cerebrovascular Function, Cognition, Mood, Physical Function and General Well-Being in Postmenopausal Women—Rationale and Study Design

**DOI:** 10.3390/nu8030150

**Published:** 2016-03-09

**Authors:** Hamish Michael Evans, Peter Ranald Charles Howe, Rachel Heloise Xiwen Wong

**Affiliations:** Clinical Nutrition Research Centre, School of Biomedical Sciences and Pharmacy, University of Newcastle, Callaghan, NSW 2308, Australia; Hamish.evans@uon.edu.au (H.M.E.); Peter.howe@newcastle.edu.au (P.R.C.H.)

**Keywords:** resveratrol, vasoactive nutrients, postmenopausal, cognition, mood, physical function, healthy ageing, cerebrovascular function, transcranial Doppler ultrasound

## Abstract

Background: This methodological paper presents both a scientific rationale and a methodological approach for investigating the effects of resveratrol supplementation on mood and cognitive performance in postmenopausal women. Postmenopausal women have an increased risk of cognitive decline and dementia, which may be at least partly due to loss of beneficial effects of estrogen on the cerebrovasculature. We hypothesise that resveratrol, a phytoestrogen, may counteract this risk by enhancing cerebrovascular function and improving regional blood flow in response to cognitive demands. A clinical trial was designed to test this hypothesis. Method: Healthy postmenopausal women were recruited to participate in a randomised, double-blind, placebo-controlled (parallel comparison) dietary intervention trial to evaluate the effects of resveratrol supplementation (75 mg twice daily) on cognition, cerebrovascular responsiveness to cognitive tasks and overall well-being. They performed the following tests at baseline and after 14 weeks of supplementation: Rey Auditory Verbal Learning Test, Cambridge Semantic Memory Battery, the Double Span and the Trail Making Task. Cerebrovascular function was assessed simultaneously by monitoring blood flow velocity in the middle cerebral arteries using transcranial Doppler ultrasound. Conclusion: This trial provides a model approach to demonstrate that, by optimising circulatory function in the brain, resveratrol and other vasoactive nutrients may enhance mood and cognition and ameliorate the risk of developing dementia in postmenopausal women and other at-risk populations.

## 1. Introduction

Dementia disproportionately affects older women (47% by the age of 85 years, compared with 31% of men). It not only destroys cognitive capabilities but also deprives individuals of their independence and places emotional and financial burdens on families, caregivers and society at large. Dementia is predicted to be the top health crisis in this century, outpacing heart disease and cancer in terms of years lost due to disability and there is currently no cure nor means of slowing its progression [1]. Furthermore, there tends to be a substantial gap between the noticeable symptoms of cognitive decline and when medical advice is sought, and a further delay before dementia is diagnosed. In Australia, it takes an average of three years from presentation of first symptoms to diagnosis [2]. As such, quality of life in the later years is likely to be sub-optimal due to impaired cognition. Cognitive impairment may also contribute to decline in physical functioning, such as activities of daily living, and is associated with increased risk of falls in the elderly [3]. Early intervention to maintain high-level cognitive function and well-being may help prolong independent living in older adults. Therefore, it is important to identify feasible lifestyle strategies to counteract cognitive decline in ageing women.

### 1.1. Estrogen, Menopause and Dementia

While women enjoy longer life expectancy, this may not be the only reason for the higher risk of dementia in elderly women. Growing evidence has shown that loss of estrogen after menopause may lead to accelerated deficits in brain function, which may lead to higher risk of dementia [4]. Ageing and many lifestyle diseases are closely related to poor blood circulatory function, including poor perfusion of brain regions. Estrogen targets blood vessels to promote blood flow in the heart, skeletal muscle and particularly in the brain to support cognitive and affective functions. Estrogen binding to estrogen receptors (ER) activates endothelial nitric oxide synthase (eNOS), modulating nitric oxide (NO)-mediated vasodilatation to meet tissue perfusion demands for optimal functioning. Importantly, ER-β are abundantly expressed in the hippocampus, a brain region which subserves learning, memory and neurodevelopmental processes [5], while ER-α are predominantly located in the basal forebrain, which coordinates information processing and attentional function [5]. Therefore deprivation of estrogen at menopause may contribute to the cognitive decline associated with neurological ageing in both executive and memory functions [4]. Executive function governs high-level cognitive functions such as time sharing, sequencing, response inhibition and perseverance, which is crucial for independent living in older adults. In the context of independent living, executive and memory functions are necessary for daily activities such as meal planning, paying bills, keeping medical appointments and ambulation [5]. It is important to acknowledge that resveratrol may also act through SIRT-1 and other mechanisms to increase NO bioavailability and boost cerebral vasodilator responses to cognitive demands [6]. However, little attention has been given to the phytoestrogenic nature of resveratrol and its potential to benefit postmenopausal women as a specific target population.

Epidemiological evidence indicates that hormone replacement therapy (HRT) halves the risk of Alzheimer’s disease in postmenopausal women [7]. However, since publication of the Women’s Health Initiative Study [8] the effects of HRT have been controversial. Five years of HRT use in late postmenopause (>70 years old) resulted in increased risk of breast cancer, stroke, heart disease and dementia, prompting many women to cease treatment and/or look for alternative treatment for their menopausal symptoms. Since the publication of these disturbing findings, there has been no follow-up investigation on whether cessation of HRT could lead to reduced risks. On the other hand, clinical evidence has shown that estrogen administration can augment mood and cognitive function postmenopausally, if commenced during early (peri) menopause rather than late menopause, due to the profound effects of estrogen in the brain [9]. Progressive impairment of cerebrovascular function is implicated in the pathogenesis of dementia [10]. Cerebrovascular responsiveness (CVR) to hypercapnic provocation (a method of assessing endothelial health in the brain) is maintained in women on HRT but is halved in non-HRT users [11]. Likewise, women with lower estrogen levels have poorer cerebral perfusion, which is further compromised during a hot flush episode [12]. HRT users exhibit higher hippocampal and temporal blood flow [measured by single positron emission tomography), which correlates with better performance on memory tasks [13], suggesting that estrogen plays a critical role in neurovascular and cognitive integrity. Impaired cerebral perfusion and executive function also contribute to poor physical function, mood disorders and higher fall rates in older adults [14]. CVR to hypercapnia was positively associated with gait speed and inversely related to number of falls in the elderly [15]. Older adults with slow gait (<0.67 m/s) had poorer CVR while performing the N-back task (a test of executive function) and had poorer test scores on executive function tasks, compared to those with faster gait [16]. Thus more attention needs to be given to the relationships between the neurovascular integrity and cognitive and physical impairments. Taken together, the available evidence strongly supports the hypothesis that cognitive, mood and physical deficits in older women may be attributed to impaired vascular function in the cerebral circulation. Thus maintenance of cerebrovascular function as part of healthy ageing may prevent the burden of cognitive and physical disability in later life. Whether cognitive and physical function deficits in postmenopausal women can be attenuated by improvement of cerebrovascular function is unknown.

### 1.2. How Could Resveratrol Benefit Postmenopausal Women?

Naturally occurring phytoestrogens such as those present in soy and red clover offer a potentially safer alternative to HRT for postmenopausal health. Genistein from soy has been shown to improve NO-mediated systemic vasodilator function in postmenopausal women [17] and thus may have the potential to enhance cerebral perfusion which may in turn improve cognition and mood. Chronic soy isoflavone supplementation has been shown to improve menopausally-related mood, memory, sleep quality and vasomotor symptoms [18]. Moreover, it has also been shown to enhance spatial working memory in young men (with low estrogen levels) [19]. However, its mechanisms of action are still unknown and conflicting evidence has discouraged clinicians from recommending soy isoflavones to menopausal women. Importantly, a large proportion of people lack the enzyme to convert daidzein to equol, which (like genistein) mimics the action of estrogen on ER-β [20], which could explain the variability in outcomes of soy interventions. In a recent study, administering 30 mg of pure (synthetic) genistein significantly reduced hot flushes [21]. Considering that cerebral perfusion is compromised during a hot flush episode [8], the improvement in menopausal symptoms may be partly attributable to enhanced cerebrovascular function. However, this hypothesis has yet to be evaluated.

Resveratrol is a phytoestrogen found in grapes, berries and nuts with multiple targeted benefits including cardiovascular and neurological, particularly attenuating learning impairment and hippocampal degeneration [22]. It may act similarly to estrogen and genistein to confer neurovascular protection by up-regulating eNOS activity, resulting in enhanced NO bioavailability and vasorelaxation [23]. Resveratrol also rapidly stimulates the mitogen-activated protein kinase signalling pathway via both ER-α and ER-β to increase eNOS activity in human endothelial cells at nanomolar concentrations that can be achieved through habitual diet, e.g., a glass of grape juice [24]. Furthermore, resveratrol can also reduce vascular tone via an endothelium-independent mechanism [23]. Given that resveratrol is structurally similar to and mimics the activity of 17-β-estradiol, it may enhance cognition by stimulating ERs [25] to increase perfusion via increased NO production. In human trials, we and others have independently demonstrated its ability to improve endothelial vasodilator function [26,27], cerebral perfusion and memory functions in older adults [28]. We found that 6-weeks daily supplementation with resveratrol (75 mg/day) resulted in a sustained improvement in flow-mediated dilatation in the brachial artery in obese but otherwise healthy older adults. There was also a non-statistical trend towards improvement in performance to the Stroop Test, suggesting that perhaps a longer duration intervention might be needed to see enhancement in cognitive function [26]. A recent study found that daily supplementation (200 mg of resveratrol with 320 mg quercetin) for 6 months in older adults resulted in greater hippocampal activity at rest, assessed by MRI, and retention of words learnt [28]. Whilst improvements in resting state activity may reflect enhancement in the integrity and functionality of the hippocampus, this augmentation does not reveal whether resveratrol can improve the efficiency of cerebral perfusion during cognitively demanding tasks, nor has it been linked to enhanced cognitive performance, mood or physical function. Thus the potential for resveratrol to maintain optimal cognitive health in postmenopausal women who are not taking HRT must now be evaluated.

The primary aim of this study is to see whether daily supplementation with resveratrol for 14 weeks can improve cognition in postmenopausal women in whom age-related endothelial dysfunction is exacerbated by estrogen deprivation. The secondary aim is to see whether improvements in cognition, mood, physical function and aspects of well-being including sleep quality, quality of life, menopausal symptoms and pain are accompanied by improvements in CVR to hypercapnia and/or to cognitive demands.

## 2. Materials and Methods

### 2.1. Study Design

A randomised, double-blind, placebo-controlled (parallel comparison) dietary intervention trial of 14 weeks duration was designed to evaluate the effects of resveratrol supplementation (75 mg twice daily) on cognitive abilities, CVR and overall well-being. The dose was based on data from an acute dose response trial in those with type 2 diabetes mellitus (manuscript in preparation), in which 75 mg resveratrol elicited maximal CVR to hypercapnia when compared with 150 mg and 300 mg doses. Assessments will be made at baseline (week 0) and at the end of the 14-week intervention phase (week 14). The trial will be conducted at the Clinical Nutrition Research Centre, University of Newcastle, in New South Wales, Australia in accordance with the Declaration of Helsinki and Principles of Good Clinical Practice as outlined by the International Conference on Harmonisation. The protocol has been approved by the University of Newcastle’s Human Research Ethics Committee (H-2015-0002; approved on 13 March 2015) and registered with the Australia and New Zealand Clinical Trials Registry on 27 March 2015 (ACTRN12615000291583).

### 2.2. Study Population

Healthy postmenopausal women from the general public residing in the Hunter region of New South Wales were invited to participate following expressions of interest to approved newspaper and radio campaigns. Each volunteer was first provided with an information statement outlining the study objectives and procedures. A health and lifestyle questionnaire that collected information pertaining to their menopausal status, medication and dietary supplement use and current perception of their cognitive status was completed by interested parties. Upon returning the questionnaire, a study investigator pre-screened volunteers via telephone to determine their potential suitability, as described in Table 1, and to invite them in for a screening/baseline visit at the research centre. They were also encouraged to consult with their medical practitioner regarding their intention to participate in this intervention prior to their first visit. Written informed consent was obtained at start of the screening/baseline visit.

Our power calculations (G*Power) [29] showed that 74 participants would give 80% chance of detecting a statistically significant difference (*p* < 0.05) of medium effect size (standardized difference = 0.67) in cognitive performance (composite score) between Resveratrol and placebo treatments. To allow for attrition, we aimed to recruit 80 women.

### 2.3. Investigational Product and Allocation

The active and placebo capsules were of identical appearance and supplied by DSM Nutritional Products Ltd, Switzerland. The active capsule comprised 75 mg of 99% pure synthetic trans-resveratrol. The placebo comprised several inert excipients: calcium hydrogen phosphate, microcrystalline cellulose, prosolv 50 and hydrated magnesium silicate. Two hundred capsules were each dispensed in sealed white opaque containers and containers were identifiable only by code numbers. An independent investigator who held the code list allocated participants to each treatment group based on the minimisation method [30], to minimise differences between groups in age and years since ceasing menses (obtained from their health and lifestyle questionnaires) prior to the scheduled screening/baseline visit. The first participant was allocated randomly. Containers allocated to volunteers who were subsequently deemed ineligible or did not attend the screening/baseline visit were reassigned and the allocation list adjusted accordingly. Blinding will be maintained until all data analysis has been completed.

### 2.4. Screening/Baseline Visit (Week 0)

Potentially eligible volunteers attended the 3-h screening/baseline visit after having refrained for at least an hour from medication, food or beverages other than water. Height, weight and waist circumference were measured followed by seated blood pressure (BP) readings to determine BP eligibility. Dementia status was then assessed using the Australian version of the Mini-Modified Mental State (3MS) examination. The 3MS is a widely used tool for screening suspected dementia; a score of less than 78 was considered impaired and necessitated exclusion from the study [31].

**Figure 1:**
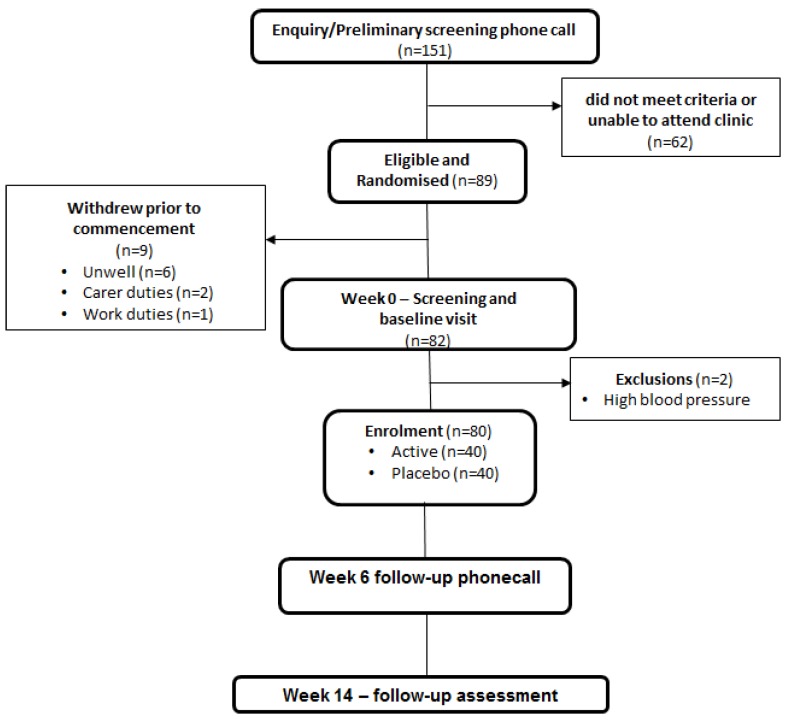
Consort diagram for study participants.

Those who met the eligibility criteria were fitted with a Transcranial Doppler (TCD) ultrasound headpiece to assess basal cerebral haemodynamics and CVR to both hypercapnic and cognitive stimuli bilaterally in the middle cerebral artery (MCA). Participant’s performances were also recorded during cognitive testing. At the conclusion of cognitive testing, participants underwent a hypercapnic assessment of CVR in the posterior cerebral artery (PCA). A physical function assessment was then carried out before a series of six paper-based questionnaires to assess mood and general wellbeing were completed to conclude the visit. Figure 1 depicts the flowchart of the participants who were enrolled in the study. It also highlights our study population size (*n* = 80, 40 per treatment group) indicating that our treatment target has been reached.

### 2.5. Intervention and Follow-up (Week 0–14)

Participants were instructed to take two capsules of resveratrol daily, preferably at the same time with meals, and to record each intake in the assigned supplement diary in order to assist with treatment compliance. If a dose was missed, participants were able to catch up the same day but discouraged from carrying over the dose to the next day. Participants were also encouraged to maintain their habitual diet throughout the intervention; however any changes in dietary supplement and/or medication intake during the intervention were to be recorded in the supplement diary. Participants were also notified to contact the study investigators by phone if they experienced any out of the ordinary side effects, became ill during the trial period or deviated from their habitual medication use. Every four weeks participants were required to complete the Pittsburgh Sleep Quality and the Menopausal Rating Scale in their supplement diaries.

A follow-up phone call will be made at week 6–8 in order to enquire about the participant’s welfare, reports of any side-effects and changes in diet, physical activity or medication use. This call will also serve to encourage compliance, with investigators checking how participants are coping with their allocated supplement and whether or not they are consuming them as instructed, and whether their questionnaires are being filled. At the end of the trial, all remaining capsules will be counted and tallied with the corresponding diary records to confirm compliance.

### 2.6. End of Intervention Visit (Week 14)

Participants will return at the end of the 14 week intervention phase for outcome assessments of clinic BP, basal cerebral haemodynamics, CVR to hypercapnia and cognitive stimuli, cognitive performance, physical function, mood and general wellbeing. These measurements will be performed in the same order as the week 0 visit; BP will be measured before the CVR to both hypercapnic and cognitive stimuli, the physical function test and mood and general well-being using the questionnaires. They will have fasted for at least one-hour and will have been instructed to refrain from consuming their supplement on the day of their visit so as to measure sustained effects of resveratrol rather than acute, as resveratrol is removed from circulation with 12 h [32].

### 2.7. Outcome Assessments

#### Clinic Blood Pressure

Participants were rested for 10 min in a seated position. Four consecutive readings of BP were taken at 2 min intervals; the first was discarded and the remaining averaged to obtain resting BP (systolic, diastolic and mean arterial pressure) and heart rate for analysis. Measurements were taken by automated oscillometry using an appropriately sized BP cuff placed over the brachial artery of the non-dominant hand (HDI Cardiovascular Profiler CR2000, Minnesota, MN, USA). BP measurements were performed by a single investigator (H.M.E), in accordance with international guidelines [33].

### 2.8. Transcranial Doppler Ultrasound Assessements

#### Basal Cerebral Haemodynamics

TCD is a non-invasive technique that utilises ultrasound to assess blood flow velocity in the brain. Using a TCD headpiece (Doppler-Box X, Singen, Germany), the MCA on both the left and right sides were isolated using the transtemporal window as this provides the least interference during insonation. The depth of insonation for MCA and PCA were between 45–60 mm and 60–70 mm respectively [34]. A 30-s of continuous recording of basal blood flow velocity in the MCA and PCA (maximum, minimum and mean) were obtained before hypercapnic provocation and before the start of each cognitive test. All basal blood flow velocities will be collated in spreadsheets and an average of the last 10 s of mean blood flow velocity (*x*) will be reported for use in CVR calculations. The pulsatility index (PI) of the MCA and PCA, reflecting stiffness in the cerebral vessels, is determined from the maximal velocity minus the minimum velocity and divided by the mean velocity obtained during basal conditions.

### 2.9. Cerebrovascular Responsiveness

Increases in blood flow velocity in the MCA and PCA reflect the extent of endothelial dilation in downstream anterior and posterior vessels respectively and thus is a good measure of CVR [35]. To assess CVR, participants inhaled a carbogen gas mixture (5% CO_2_, 95% O_2_) through a two-way non-rebreathing mouthpiece for 180 s, resulting in an acute increase in blood flow velocity. The TCD recorded bilateral beat-to-beat mean blood flow velocities during the hypercapnic provocation. Our group has shown great reproducibility and thus the procedure was only repeated once for reliability with a 2 min interval for washout.

The TCD headpiece was kept in position throughout the cognitive test battery to assess CVR to cognitive stimuli in the MCA and was recorded during each cognitive task

Data will be stored on the computer hard drive until subsequent analysis. Each task, including hypercapnia, recordings would be smoothed and analysed in TableCurve™ using data spline estimation with Loess at 20% for hypercapnic assessment and 10%, for cognitive tasks, (TableCurve 2D by Systat Software Inc., San Jose, CA, USA) to determine the peak increase in mean flow velocity (y). CVR is calculated as a percentage using the equation$\;\frac{y-x}{x}\times 100$.

### 2.10. Cognitive Performance

The neuropsychological test battery consisted of four tests, The Rey Auditory Verbal Learning Test (RAVLT) [36], the Cambridge Semantic Memory Battery [37], the Double Span [38] and the Trail Making Task (TMT) [39]. The composition of the test battery was chosen such that it would assess those cognitive domains known to be negatively affected by reductions of circulating estrogen in women, namely semantic [40], verbal [41] and visual spatial working memory [42,43] as well as executive function, shown to decline with age irrespective of gender [44]. Importantly these tests also reflect the ability to perform everyday tasks with a decline indicating a loss of independence in these women. Table 2 outlines these tests, describing the cognitive domains targeted, the procedure and scoring profiles.

### 2.11. Assessment of Mood

Participant’s mood states were assessed using two different indexes, the Profile of Mood States (POMS-V2) and the Center for Epidemiological Studies Depression Scale (CES-D). The POMS questionnaire assessed how the participant was feeling over the last week including the day of the visit through 65-adjectives that the participant rated on a 5-point Likert scale (1 being “not at all” and 5 being “extremely”). The POMS Standard has proven to be an excellent measure of mood states and their fluctuations [45]. The CES-D scale is a commonly used tool to characterize depressive symptoms in the general population [46] and the sensitivity ideal for detecting depression in postmenopausal women [47]. The 20-item scale measures major depressive symptomatology of participants, including depressive mood, feelings of guilt and worthlessness, psychomotor retardation, loss of appetite and sleep disturbances.

### 2.12. Physical Function Assessment

The physical function test battery is a validated measure of functional performance of daily living activities in community living older adults consisting of nine tasks [48]. For each task, the time taken to complete was recorded. Participants were required to write a legible sentence, mimic eating by picking up kidney beans with a teaspoon from a bowl and placing them in another while they remain stationary, lifting a heavy book from waist level and placing on a cabinet at shoulder level while remaining seated, put on and remove a coat (A standard lab coat was used to maintain consistency), pick up a 50 cent coin from the floor, pivot 360°, walk 15 metres and climb flights of stairs. Assistive devices are permitted in the locomotive tasks. Higher scores equated to better performances.

### 2.13. Assessment of Overall Well-Being

Overall wellbeing was assessed through a series of four different questionnaires, designed to assess varying aspects of a participant’s general living. Participant’s pain symptomatology was assessed through the Short-form McGill Pain questionnaire [49]. Sleep quality was assessed through the Pittsburgh Sleep Quality Index [50], Menopausal symptoms were assessed using the Menopausal Rating Scale (MRS) as this has been shown to be effective in measuring treatment effects on quality of life across the full range of severity of complaints in ageing women [51] and the participants own perception of their physical and mental health were also recorded using the Short-form 36 (SF-36) Health Survey, a validated questionnaire in menopausal women [52].

### 2.14. Statistical Analysis

The primary outcome is the effect of resveratrol supplementation *versus* placebo on cognitive performance, determined using a composite score of all memory tests. Treatment by time effects will be determined by analysis of variance (ANOVA), or analysis of covariance (ANCOVA) in the event of interacting variables. the process of subject allocation by minimisation will have balanced age and years since cessation of menses between groups; however other baseline characteristics such as clinic BP, basal PI, BMI, 3MS score and years of education will be considered covariates if they were significantly correlated with the primary outcome.

Treatment changes in CVR to hypercapnia and/or cognitive stimuli, improvements in physical function, mood states and quality of life will be assessed using linear regressions with false rate discovery adjustments used to minimise type 1 error. In addition, to explore the potential benefits of early intervention, the magnitude of improvement following resveratrol supplementation will be compared in early (<10 years) and late (>10 years) postmenopausal women. Statistical significance for the primary outcome is set at *p* < 0.05 and false discovery rate will be applied to secondary outcomes. Statistical analyses will be performed using SPSS version 21.0 (SPSS by IBM Inc., Chicago, IL, USA).

## 3. Conclusions

We have identified postmenopausal women as a vulnerable population at increased risk of cognitive decline and dementia who may benefit from supplementing their diet with resveratrol. This paper provides a rationale for investigating the effects of resveratrol supplementation on cognitive performance and mood in postmenopausal women based on our hypothesis that resveratrol can augment both by enhancing cerebrovascular perfusion. According to our hypothesis, any improvements in cognition will be secondary to improvements in CVR; however, the technical limitations of obtaining reliable TCD ultrasound signals, particularly in older women, did not warrant the inclusion of cerebrovascular function as the primary outcome as recruitment may be limited. Nonetheless it provides a detailed study design and protocol for undertaking this investigation, utilising TCD as a novel approach to evaluate impacts of supplementation on CVR. Additionally, through this paper we have outlined a model approach for which we can establish a portfolio of evidence for the efficacy of not only resveratrol but other vasoactive nutrients in maintaining optimal circulatory function to ameliorate the risk of developing dementia.

Given that resveratrol supplements are readily available over-the-counter to consumers, the anticipated outcomes will offer a non-pharmaceutical approach for managing menopause-related symptoms and counteracting accelerated cognitive and physical decline as well as mood disorders in women postmenopausally, which can be readily implemented by clinicians and the public.

## Figures and Tables

**Table 1:** Eligibility criteria for the study.

Inclusion Criteria
Aged 45–85 years Postmenopausal (self-reported cessation of menses for more than 6 months) Stable current medication (unlikely to change for duration of the study)
Exclusion criteria
Currently taking HRT or alternative hormone therapy for the relief of postmenopausal symptoms or have done so in the last 6 months Breast or cervical cancer Other serious disease, such as cardiovascular disease, other cancer, kidney or liver disease or neurologic condition Smokers or currently on nicotine therapy Using insulin or warfarin therapy Clinically significant depression Currently consuming more than 4 alcoholic beverages a day Changed medication in the 3 months prior to study enrolment Clinic BP > 160/100 mmhg (determined at screening) Suspected dementia (determined at screening)

**Table 2:** Components of the Neuropsychological test battery.

Cognitive Test & Domains	Procedure	Scoring
**RAVLT (immediate and long-term verbal memory)**	*Immediate:* The participants were required to listen to an electronic audio containing 15 nouns (list A) presented at a rate of 1 word/2 s. After the audio was played, participants were required to verbally recall the words irrespective of order (trial A1). The list was presented a further four times (trial A2-A5). A second interference list (list B) was presented in the same manner (trial B6). Immediately afterward trial B6 participants were instructed to recall all the words from list A without prompt (trial A7).	Scaled scores = raw scores − errors
		Learning = scaled total (Trials A1-A5).
		Proactive memory = scaled Trial B6.
	*Delayed:* participants performed other tasks for 20 min before being required to recall all 15 words from list A (trial A8). Participants were then provided with a sheet containing all 15 words from both list A and B as well as 20 words similar in either phonology or semantic terms (total 50 words) and required to correctly identify words from both list A and list B.	
		Retroactive interference = scaled trial A7-scaled trial A5.
	For each trial, number of errors was also recorded.	
		Delayed recall = scaled trial A8.
		Recognition = Z-score list A + Z-score list B + Z-score Errors
**Cambridge Semantic Memory Battery (semantic memory)**	*Category fluency:* Participants were given 1 min to give as many items within a given category as they could. 8 categories were used, separated into living (animals, fruits, Birds, breed of dogs) and non-living (household items, tools, vehicles, types of boats)	Number of correct responses overall.
		As per category fluency.
	*Letter fluency:* Participants were given 1 min to give as many words as they could starting with a given letter. Letters used were F, A and S. Proper nouns were excluded.	
	*Naming*: participants were required to correctly name 64 images of common living and non-living items, presented one by one.	
		As per category fluency.
	*Category Comprehension:* Participants were read the names of 64 items and required to correctly identify each from groups of images closely related to the stimuli.	
	*Semantic Association (camel and cactus):* Participants were shown an item and must correctly identify, from a separate group of 4 items, the item that is best associated with the first.	
		As per category fluency.
		As per category fluency.
**Double Span Task (visuospatial working memory)**	14 different objects were used in this task. For each task, a random number of objects (max = 5) displayed in random positions on 4 × 4 grid. Participants were prompted to either recall positions (point to position of each object in the order they were presented) or positions and names (point to the position of each object in the order they were presented saying the name of each object verbally) in an empty grid after a 5 s delay.	“Positions” = number of each correct position in order.
		“Position and names” as per “positions” with an additional mark for each correct name in order.
		Marks were then tallied for an overall score.
**Trail Making Task (executive function and processing speed)**	*Trial A:* Participants were required to draw a continuous line connecting 25 numbers spread randomly across the page in ascending order.	Trial A and B = Time taken, errors
	*Trial B:* Participants were required to draw a continuous line alternating between numbers and letters (1,a,2,b,3,c, *etc.*) spread randomly across the page	Interference = time taken (trial B/Trial A)

## Abbreviations

The following abbreviations are used in this manuscript:

ER: estrogen receptors
eNOS: endothelial nitric oxide synthase
NO: nitric oxide
HRT: hormone replacement therapy
CVR: cerebrovascular responsiveness
BP: blood pressure
3MS: Mini Modified Mental State Examination
TCD: transcranial Doppler
MCA: middle cerebral artery
PCA: posterior cerebral artery
PI: pulsatility index
RAVLT: Rey Auditory Verbal Learning Test
TMT: Trail Making Task
POMS: Profile of Mood States
CES-D: Center for Epidemiological Studies Depression Scale
MRS: menopausal rating scale
SF-36: Short-form 36
ANOVA: analysis of variance
ANCOVA: analysis of covariance
